# Sodium butyrate does not protect spinal motor neurons from AMPA-induced excitotoxic degeneration *in vivo*

**DOI:** 10.1242/dmm.049851

**Published:** 2023-10-13

**Authors:** Mara Prior-González, Rafael Lazo-Gómez, Ricardo Tapia

**Affiliations:** División de Neurociencias, Instituto de Fisiología Celular, Universidad Nacional Autónoma de México, Circuito exterior s/n, Ciudad Universitaria, Coyoacán, Mexico City 04510, Mexico

**Keywords:** Motor neuron, Paralysis, Histone deacetylase, Excitotoxicity, Amyotrophic lateral sclerosis

## Abstract

Motor neuron (MN) loss is the primary pathological hallmark of amyotrophic lateral sclerosis (ALS). Histone deacetylase 4 (HDAC4) is one of several factors involved in nerve–muscle communication during MN loss, hindering muscle reinnervation, as shown in humans and in animal models of ALS, and may explain the differential progression observed in patients with ALS – rapid versus slow progression. In this work, we inhibited HDAC4 activity through the administration of a pan-histone deacetylase inhibitor, sodium butyrate, in an *in vivo* model of chronic spinal MN death induced by AMPA-mediated excitotoxicity. We infused AMPA into the spinal cord at low and high doses, which mimic the rapid and slow progression observed in humans, respectively. We found that muscle HDAC4 expression was increased by high-dose infusion of AMPA. Treatment of animals with sodium butyrate further decreased expression of muscle HDAC4, although non-significantly, and did not prevent the paralysis or the MN loss induced by AMPA infusion. These results inform on the role of muscle HDAC4 in MN degeneration *in vivo* and provide insights for the search for more suitable therapeutic strategies.

## Teaser

**Summary:** In rats with chronic spinal motor neuron death induced by AMPA-mediated excitotoxicity, HDAC4 inhibition through administration of sodium butyrate does not ameliorate paralysis or muscle pathology.

## INTRODUCTION

Spinal motor neurons (MNs) are specialized neurons located in the anterior horn of the spinal cord's gray matter that integrate and transmit commands from the central nervous system to their targets in the periphery, the skeletal muscle cells. Neuromuscular junctions (NMJs) are the specialized synapses between MN axon terminals and skeletal muscle cells. Besides being involved in acetylcholine-mediated neurotransmission, NMJs are the loci at which MNs and skeletal muscle establish bi-directional communication paramount for the development and maintenance of MN and skeletal muscle cell types, for example, through trophic factor secretion (Wu et al., 2010).

MN disorders are a group of neurodegenerative diseases in which death occurs in 50% of patients within the first 1-2 years after diagnosis; selective MN loss is the patients’ common pathological hallmark (Logroscino et al., 2018). MN death results in muscle weakness and paralysis by the progressive loss of NMJs and, consequently, the inability to recruit skeletal muscle (Blizzard et al., 2015). Amyotrophic lateral sclerosis (ALS) is the most common type of adult-onset MN disorder, and the rate of MN loss varies among ALS patients. Indeed, nearly 14% of ALS patients develop a long-term illness, surviving >10 years (Mateen et al., 2010); compensatory reinnervation by remaining healthy MNs may explain this prolonged survival.

Although the mechanisms behind the selective MN degeneration in ALS have not been conclusively elucidated, glutamate-mediated excitotoxicity is one of the most widely accepted mechanisms (Tovar-y-Romo et al., 2009). This is supported by the fact that there are only two drugs approved to slow disease progression (riluzole and ederavone), both of which ameliorate excitotoxic neuronal death (Abe et al., 2014; Albo et al., 2004), although they have very limited efficacy and prolong survival for only a few months. However, several lines of evidence have suggested that MN degeneration could arise from defects in skeletal muscle signaling and metabolism (‘die-back hypothesis’) (Loeffler et al., 2016). For example, it has been shown that muscle overexpression of Nogo-A (reticulon), an axon regeneration inhibitor, correlated with denervation severity in ALS patients (Bruneteau et al., 2015). Whether MN degeneration begins within the central nervous system and then is perpetuated by pathological processes in the skeletal muscle, or the reverse, remains to be clarified. Elucidating this could provide guidance on the development of better therapeutic strategies.

Histone deacetylases (HDACs) are a family of 18 zinc-dependent enzymes that remove acyl groups from lysine residues in proteins, and are involved in regulating cell signaling, gene expression and metabolism (Kouzarides, 2007). HDAC4, a subclass IIa HDAC, is ubiquitously expressed in mammalian tissues, including skeletal muscle and MNs, and is characterized by its shuttling between cytoplasm and nucleus (Lazo-Gómez et al., 2013). HDAC4 is strongly involved in NMJ maintenance, as levels of this HDAC were inversely correlated with skeletal muscle reinnervation in ALS patients with short-term illness (Bruneteau et al., 2013). In support of this finding, in SOD^G93A^ mice, an animal model of ALS, selective HDAC4 deletion in skeletal muscle improved reinnervation (Williams et al., 2009). It has been demonstrated that changes in HDAC4 expression within skeletal muscle cells are triggered by a lack of MN activity. Indeed, MN degeneration induces HDAC4 expression and its nuclear accumulation in skeletal myocytes, specifically in regions close to the NMJ (Cohen et al., 2007). Blocking HDAC4 nuclear export initiates the DACH2/myogenin/nicotinic acetylcholine receptor-dependent signaling cascade (Cohen et al., 2007), inactivates myocyte-specific enhancer factor 2 (MEF2) (Wang et al., 2014) and downregulates fibroblast growth factor binding protein 1 (FGFBP1) (Williams et al., 2009); these changes ultimately result in inhibition of NMJ establishment and maturation and disruption of muscle tissue remodeling.

Taking this evidence into account, we hypothesized that pharmacologically inhibiting HDAC4 would ameliorate paralysis and prevent MN degeneration through the daily administration of the pan-HDAC inhibitor, sodium butyrate (NaBut), in a well-established *in vivo* model of spinal chronic excitotoxicity achieved by the continuous infusion of AMPA with osmotic minipumps (Tovar-y-Romo et al., 2007).

## RESULTS

### AMPA infusion alters skeletal muscle HDAC4 expression *in vivo*

**Fig. 1.**
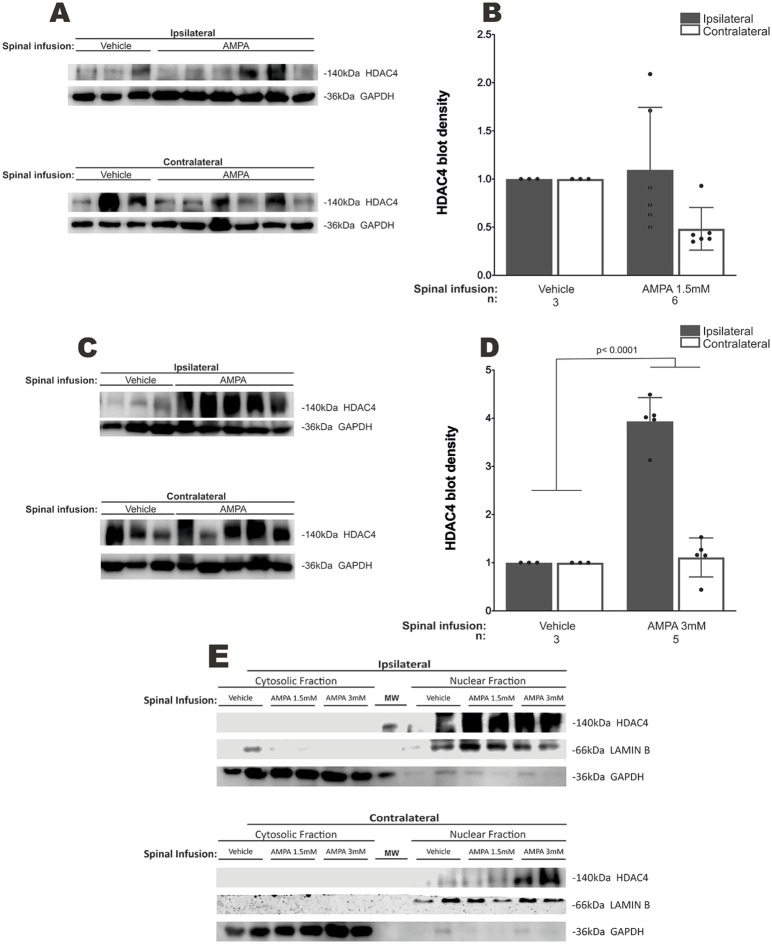
**HDAC4 expression in skeletal muscle of rats subjected to chronic spinal excitotoxicity.** (A,C) HDAC4 immunoblots from ipsilateral and contralateral biceps femoris muscles from animals spinally infused with vehicle (*n*=3) or 1.5 mM AMPA (*n*=6) for 10 days (A) or with 3 mM AMPA (*n*=5) for 7 days (C). (B,D) Bar graphs show quantifications of relative blot intensity in A and C, respectively. (E) HDAC4 immunoblots from cytosolic and nuclear fractions from ipsilateral and contralateral biceps femoris muscles, (*n*=2 for each group). *n*, number of animals. Mean±s.e.m., one-way ANOVA with Bonferroni post-hoc test.

Spinal infusion of AMPA *in vivo* with osmotic minipumps results in chronic excitotoxicity, MN degeneration and paralysis, and the rate of progression of these changes is directly proportional to AMPA dose (Tovar-y-Romo et al., 2007), in a manner analogous to slowly and rapidly progressing transgenic ALS murine models and to short- and long-term ALS patients (Ramírez-Jarquín and Tapia, 2016; Santa-Cruz et al., 2016). To further characterize our model of AMPA-mediated MN degeneration *in vivo*, we aimed to confirm that denervation induces skeletal muscle HDAC4 overexpression, as has been previously reported in ALS patients (Bruneteau et al., 2013). Therefore, we infused two different AMPA dose regimes, 1.5 mM for 10 days (low-dose) and 3 mM for 7 days (high-dose), directly into lumbar ventral horn spinal cord tissue, as previously described (Tovar-y-Romo et al., 2007). These two regimes were intended to simulate short- and long-term illness, respectively. At the end of the experimental timeline, animals displayed significant hindlimb skeletal muscle denervation and atrophy (Fig. S1), resulting in bilateral paralysis. Low-dose AMPA infusion (1.5 mM) resulted in non-significant decreasing levels of HDAC4 protein in the skeletal muscle tissue contralateral to the infusion side (1.09±0.26 versus normalized control in ipsilateral side, *P*>0.9999) (Fig. 1A,B), whereas high-dose AMPA infusion (3 mM) caused a ∼4-fold increase in HDAC4 protein expression in the ipsilateral biceps femoris muscle (3.93±0.22 versus normalized control in ipsilateral side, *P*<0.0001) (Fig. 1C,D). Also, HDAC4 expression was restricted to the nucleus in biceps femoris muscle under vehicle and AMPA spinal infusion conditions (Fig. 1E).

### NaBut administration does not increase histone 3 K9,14 acetylation

**Fig. 2.**
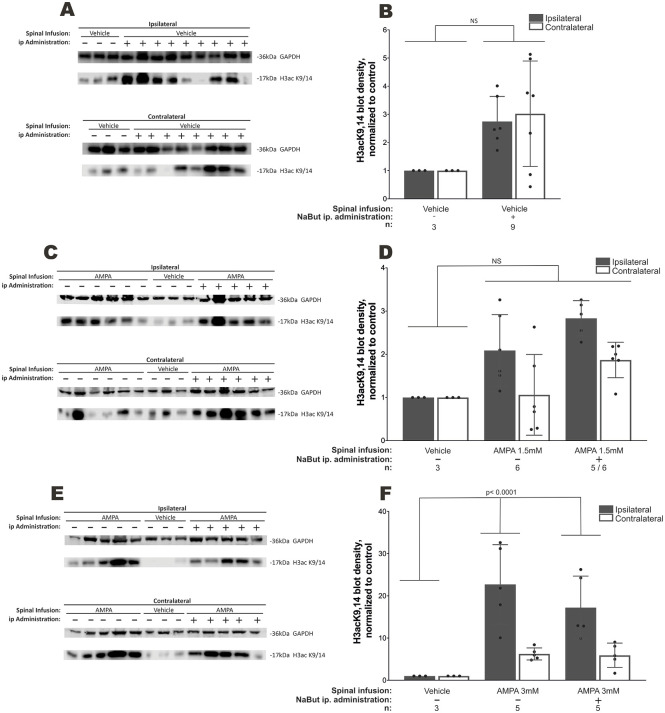
**Changes in H3K9,14 acetylation in skeletal muscle induced by sodium butyrate (NaBut) and chronic spinal excitotoxicity.** (A,C,E) H3K9,14 acetylation immunoblots after daily NaBut administration in rats spinally infused with vehicle (control, *n*=9) (A), 1.5 mM AMPA (*n*=5 for ipsilateral side and *n*=6 for contralateral side) (C) and 3 mM AMPA (*n*=5) (E) versus rats with vehicle administered intraperitoneally (*n*=3). (B,D,F) Bar graphs show quantifications of relative blot intensity in A, C and E, respectively. *n*, number of animals. Mean±s.e.m., one-way ANOVA with Bonferroni post-hoc test. i.p., intraperitoneal; NS, not significant.

As HDAC4 activity is involved in NMJ maintenance, we hypothesized that inhibiting this HDAC, with the pan-HDAC inhibitor NaBut, could ameliorate denervation in our model of AMPA-induced MN death. Because NaBut is quickly metabolized, we administered the highest possible dose [500 mg/kg/day intraperitoneal (i.p.) administration for the total length of the experiment] to achieve sustained inhibition of all HDACs. We found that acetylation of histone 3 at lysines 9 and 14 (H3K9,14), both targets of HDAC4, were increased in the ipsilateral and contralateral skeletal muscles after NaBut administration in spinally infused vehicle animals, although the changes were not statistically significant (2.74±0.36 versus normalized control in ipsilateral side, *P*>0.9999) (Fig. 2A,B). In low-dose AMPA-treated animals, acetylation increased 2-fold only in the ipsilateral muscles, and NaBut administration increased this acetylation almost 3-fold (2.08±0.33 and 2.83±0.18, versus normalized control in ipsilateral side, respectively, *P*>0.9999) (Fig. 2C,D). High-dose AMPA infusion resulted in further increased H3K9,14 acetylation levels versus those of normalized control in the ipsilateral side (*P*<0.0001), although there were no differences between vehicle and NaBut-administered animals (22.72±4.20 versus 17.22±3.33, *P*>0.9999) (Fig. 2E,F).

### NaBut administration does not ameliorate AMPA-induced paralysis

**Fig. 3.**
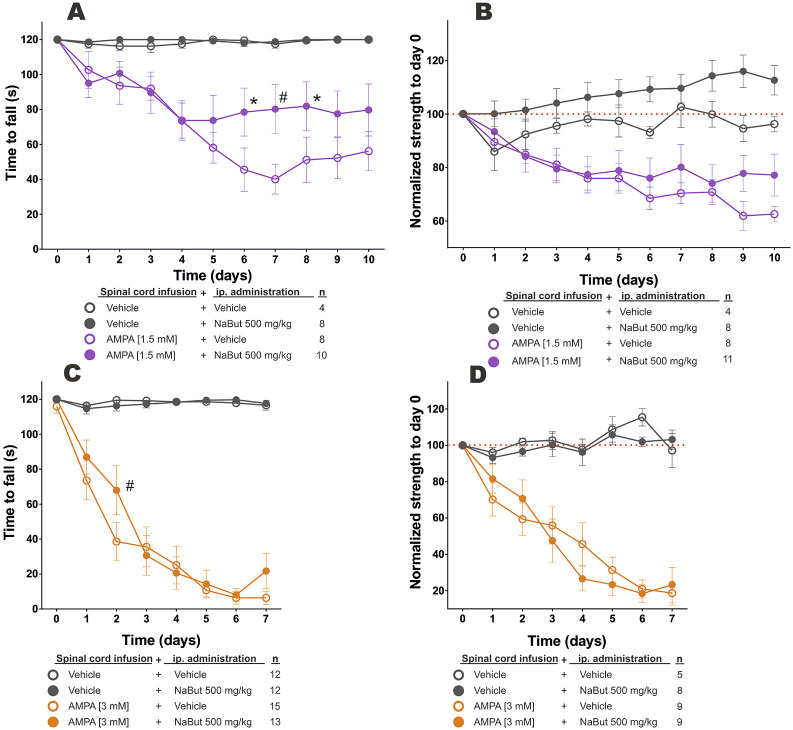
**Motor behavior performance in animals treated with NaBut under chronic spinal excitotoxicity.** (A-D) Time to fall (in s) from the rotarod and normalized hindlimb strength to day 0 in low-dose (A,B, respectively) and high-dose (C,D, respectively) AMPA-treated rats. *n*, number of animals. Mean±s.e.m., two-way ANOVA with Bonferroni post-hoc test*.* *P<0.05 and ^#^*P*<0.005 versus AMPA spinally infused and vehicle administered intraperitoneally group, respectively.

Control animals did not have any changes in hindlimb motor performance, as determined by rotarod and hindlimb strength assessment tests, throughout the experiment (7 or 10 days, as corresponding to the AMPA dose regime). Animals administered with NaBut had similar results in both tests (Fig. 3, filled gray circles).

Low-dose AMPA infusion caused slowly progressing hindlimb muscle atrophy and paralysis; these animals were followed for 10 days, at which point the time to fall from rotarod reached no longer than ∼56 s and strength fell to ∼60% of baseline values (Fig. 3A,B). NaBut administration in low-dose AMPA infusion animals slightly improved hindlimb movement, which might explain the longer time to fall from the rotarod, with differences compared to vehicle administration in low-dose AMPA infusion animals statistically significant at day 6, 7 and 8 (78.5±13.69 s versus 45.5±12.43 s at day 6, 80.2±14.02 s versus 40.12±8.55 s at day 7, and 81.9±13.94 s versus 51.12±13.00 s at day 8; *P*=0.0285, *P*=0.0050, *P*=0.0465, respectively) (Fig. 3A). No changes were observed in the hindlimb strength test (Fig. 3B).

High-dose AMPA infusion resulted in rapidly progressing hindlimb muscle atrophy and paralysis. The animals in this group were followed for 7 days because at this point most animals had complete hindlimb paralysis and were unable to perform the motor tests. In the rotarod test, there was a faster decline in performance in high-dose AMPA infusion animals; for example, by day 2, the time to fall had decreased to 38.53±11.02 s (versus 67.92±13.99 s in high-dose AMPA NaBut-treated animals; *P*<0.0050) (Fig. 3C). Strength also displayed a faster rate of decline, and, by day 4, animals had ∼45% of baseline values (Fig. 3D). NaBut administration with this condition did not result in further changes in any of the motor tests (Fig. 3C,D).

### NaBut does not prevent excitotoxic MN death

**Fig. 4.**
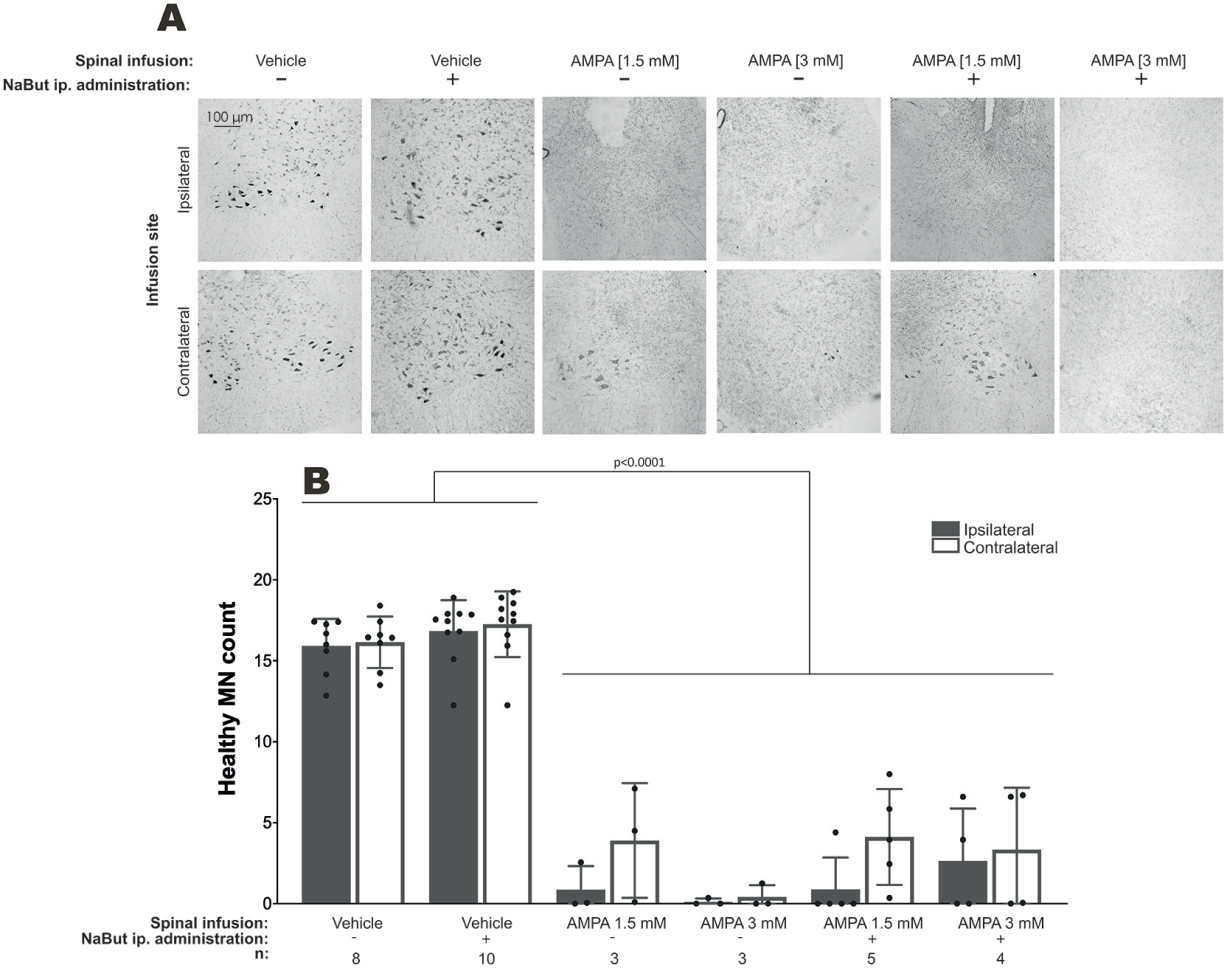
**Spinal motor neuron (MN) quantification in animals subjected to chronic excitotoxicity.** (A,B) Representative low-magnification micrographs of healthy Nissl-stained lumbar spinal cord (L4) ventral horn MNs (A) and quantification (B) at the end of the experiments. *n,* number of animals. Mean±s.e.m., one-way ANOVA with Bonferroni post-hoc test.

As shown in previous studies (Lazo-Gomez and Tapia, 2017), low- and high-dose AMPA spinal infusions caused complete MN loss on the ipsilateral side to the infusion, but only the high-dose AMPA spinal infusion resulted in total neurodegeneration on the contralateral side to the infusion (Fig. 4A). This MN degeneration was accompanied by NMJ denervation and biceps femoris muscle pathological changes (including muscle fiber atrophy and hypertrophy; Fig. S1). Spinally infused vehicle solution did not cause any change in spinal cord histology or in MN number. NaBut administration was not neuroprotective in any experimental condition tested, although it was not neurotoxic in animals spinally infused with vehicle solution, as assessed by spinal cord histology or MN number (Fig. 4A,B).

## DISCUSSION

ALS is the most common MN disorder, and currently there are no effective therapeutic strategies to halt disease progression. The rational development of effective therapeutic strategies must be based on understanding the pathophysiological mechanisms underlying MN degeneration. Some of the molecular features of spinal MN make them highly vulnerable to excitotoxicity (Brockington et al., 2013; Gregory et al., 2020; Shaw and Eggett, 2000), and several lines of evidence in the SOD1^G93A^ transgenic model of ALS reinforce the idea that excitotoxicity is a fundamental mechanism of MN degeneration (Bonifacino et al., 2019; Jiang et al., 2017; Ohgomori et al., 2017). Therefore, excitotoxicity is one of the most accepted mechanisms involved in neurodegeneration. Additionally, MN loss results in NMJ denervation, nerve–muscle communication failure and, as a consequence, weakness and paralysis. In support, Blizzard et al. (2015, 2016) previously reported that spinal chronic excitotoxicity, although elicited through the infusion of kainic acid in mice, resulted in the same sequence of neuromuscular pathology – MN loss, NMJ denervation and paralysis – which is in agreement with our findings. However, whether MN degeneration in ALS begins centrally or peripherally has not been fully elucidated. Indeed, another possible mechanism for MN degeneration is communication failure between MN and muscle cells.

For example, muscle HDAC4 expression increases the most as a result of neural–muscle communication failure, in contrast to other HDACs (such as HDAC5, HDAC6, HDAC7 and HDAC9) (Cohen et al., 2007), and this has been observed in the SOD1^G93A^ transgenic mice (Buonvicino et al., 2018). Also, in patients with ALS, correlation between muscle HDAC4 protein expression and disease progression and survival was reported (Bruneteau et al., 2013), providing further support to the role of HDAC4 in nerve–muscle communication. These results suggest that increased muscle HDAC4 protein levels are associated with rapid disease progression and worse outcomes in ALS. However, this idea was not supported by a study in which conditional knockout of muscle HDAC4 in double SOD1^G93A^ transgenic mice had no effect on progression and survival compared with those of the single SOD1^G93A^ transgenic animals (Pigna et al., 2019). These contrasting results might be due to the models and techniques used to modulate HDAC4 expression or activity and need further clarification.

In this work, we found that the expression of muscle HDAC4 protein in an excitotoxic MN degeneration model induced by AMPA infusion in the spinal cord was only increased in the high-dose group (3 mM), which is analogous to rapidly progressing ALS. However, muscle HDAC4 expression was unmodified in the low-dose AMPA-treated animals (1.5 mM), similar to slowly progressing ALS (Bruneteau et al., 2013). These changes might not be explained by NMJ denervation and subsequent muscle pathology, as both the low-dose and high-dose AMPA-treated animals displayed these alterations. Indeed, at the end of the experiments, both low-and high-dose AMPA-treated animals' hindlimbs had nearly the same degree of paralysis. However, the discrepancy in the changes of muscle HDAC4 expression might be explained by different kinetics of NMJ loss. Another explanation could be that the biceps femoris, the muscle evaluated in this study, is highly adaptable and is able to transform the metabolic phenotype of its fibers according to energetic demands, and that transformation may confer this muscle resistance to degeneration (Dahmane et al., 2006). We also found that HDAC4 expression was restricted to the nucleus of skeletal cells, and this result correlates with previous reports that HDAC4 nuclear accumulation results from MN degeneration, thus contributing to NMJ deterioration (Cohen et al., 2007). In agreement, fast glycolytic muscle fibers are the first and most susceptible to degeneration in early ALS stages (Atkin et al., 2005; Cohen et al., 2007), and HDAC4 has been involved in the denervation-dependent glycolysis failure in these fibers through muscle-specific enolase and phosphofructokinase gene repression (Tang et al., 2009). This evidence, however, does not clarify whether the pathological process in ALS begins in MNs or in muscle tissue.

Because there are no selective HDAC4 inhibitors, we decided to work with NaBut, an HDAC pan-inhibitor that easily penetrates the blood–brain barrier and that has shown potential in several neurodegenerative diseases and models (Chuang et al., 2009), administered systemically. Considering that NaBut is quickly metabolized and may cause oxidative stress at high doses, we decided to work with a daily i.p. dose, in accordance with pharmacokinetic studies, in order to elicit changes in histone acetylation but avoid most stress-like effects attributed to NaBut (Gagliano et al., 2014; Kwon and Houpt, 2010). We hypothesized that if nerve–muscle communication failure began in the periphery – muscle tissue – NaBut would at least ameliorate some of the behavioral and histological changes induced by AMPA in the spinal cord. However, NaBut did not increase the acetylation marks at lysines 9 and 14 of histone 3 (H3acK9,14), two residues known to be deacetylated by HDAC4 (Wang et al., 2014), in vehicle- and AMPA-treated animals, regardless of the dose. Interestingly, we observed that the excitotoxic conditions themselves resulted in increased H3acK9,14, which could indicate HDAC4 inhibition. This finding may be due to intrinsic mechanisms aimed at halting potential muscle HDAC4 deleterious activity. In fact, miRNA-206, a skeletal muscle microRNA key for NMJ maintenance and shown to promote recovery in ALS transgenic animal models, acts through HDAC4 translational inhibition (Williams et al., 2009).

Systemic NaBut administration, and hence HDAC4 inhibition, did not ameliorate MN excitotoxic degeneration, NMJ denervation and paralysis as would be expected if muscle HDAC4 contributed to nerve–muscle dysfunction. This finding might add to the evidence suggesting that the pathological process in nerve–muscle communication failure in ALS and other neuromuscular disorders begins in MNs (Eisen, 2021; Fogarty, 2019). For example, in a murine model of spinal muscular atrophy, a disorder characterized by chronic MN loss, NaBut did not prolong the lifespan or prevent MN death (Butchbach et al., 2016). By contrast, NaBut administration effectively hindered muscle atrophy in an acute denervation model by sciatic nerve crushing (Walsh et al., 2015). These contrasting results might be explained by the kinetics of muscle denervation – slowly progressing MN loss might put into motion other molecular mechanisms not susceptible to NaBut effects. Furthermore, because NaBut is a non-specific HDAC pan-inhibitor, the potential beneficial effects of other HDAC activities could have been precluded. In agreement, early inhibition of HDAC1/2 (Brügger et al., 2017) or of HDAC5 (Cho and Cavalli, 2012) after sciatic nerve crush promotes axonal regeneration and remyelination either by accelerating Schwann cell conversion into repair cells or by allowing microtubules to be in a more dynamic state in the axonal growth cone. These mechanisms might explain the initial, but transitory, behavioral recovery observed in SOD1^G93A^ transgenic mice administered MC1568, an HDAC4/5/6 inhibitor (Buonvicino et al., 2018). Indeed, this might explain the preservation of the behavioral performance of low-dose AMPA-treated animals administered NaBut (time to fall was greater than ∼60% and hindlimb strength was greater than ∼75% of baseline values from day 4 onwards), which was different from the performance of animals subjected to low-dose excitotoxic conditions. Interestingly, 4-phenylbutyrate, a closely related compound to NaBut that is also an HDAC inhibitor, has been shown to promote survival in two models of chronic MN loss (Butchbach et al., 2016; Petri et al., 2006), while the combination of phenylbutyrate and taurursodiol significantly reduced functional decline in humans with ALS (Paganoni et al., 2020) and recently received U.S. Food and Drug Administration (FDA) approval (Aschenbrenner, 2023). The reasons behind these differences have not been completely elucidated.

In summary, we here show that excitotoxic chronic MN loss *in vivo* induces HDAC4 expression in skeletal muscle and that inhibition of HDAC4 activity does not prevent MN loss or paralysis.

## MATERIALS AND METHODS

### Animals

Male Wistar rats (260-310 g) were used in all experiments. The animals were housed in individual cages under standard room temperature and under light/dark-controlled cycles, with access to food and water *ad libitum*. They were handled in accordance with the rules for Research in Health Matters (Mexico) and under institutional approval of the animal care committee (RTI21-14).

### Treatment, osmotic minipump preparation and experimental design

α-Amino-3-hydroxyl-5-methyl-4-isoxazole-propionate (AMPA; Abcam) was infused through an Alzet osmotic minipump (Model 2004; capacity, ∼234 µl; flow rate, 0.23 μl/h) directly into the spinal cord tissue. Osmotic minipumps were filled with PBS (for control groups) or with 1.5 mM (low-dose) and 3.0 mM (high-dose) AMPA dissolved in filtered PBS, and were incubated in filtered isotonic saline solution at 37°C for 48 h before surgical implantation to achieve a constant flow rate. NaBut (Sigma-Aldrich) at a dose of 500 mg/kg (0.9 M), or PBS (as control), was administered intraperitoneally. Each batch of NaBut solution was freshly prepared in sterile filtered PBS. Intraperitoneal administration was performed daily after motor behavior assessment for 6 days or 9 days after minipump surgical implantation (see below). The combination of osmotic minipump infusion and i.p. administration yielded six groups: control (vehicle spinally infused and vehicle administered intraperitoneally), control+NaBut, AMPA low-dose or AMPA high-dose+vehicle, and AMPA low-dose or AMPA high-dose+NaBut. Every AMPA low-dose or high-dose group was surgically implanted and studied at the same time as the other groups.

### Surgery minipump implantation

Osmotic minipump surgical implantation was carried out as described by Tovar-y-Romo et al. (2007) with minor modifications. Briefly, rats were anesthetized with isoflurane 5.0%-carbogen (95% O_2_/5% CO_2_) mixture for 4 min at 5 l/min and placed in a stereotaxic rat spinal unit; anesthesia was maintained at a constant rate of 1-2 l/min. Rats were shaved in the back in the lumbar region, the skin was disinfected, and a 4 cm longitudinal incision was made. Muscle fascia was removed, muscle tissue surrounding vertebrae L3-L4 were retracted, and L4 vertebral spinous process was removed with a dental drill to clearly visualize both vertebral laminae. On the left lamina, a hole was drilled, and a stainless-steel screw was fixed to support the implant (see below); on the right side, a ∼2 mm diameter laminectomy was performed until spinal cord visualization. The meninges were carefully removed and then a cannula was vertically inserted (perpendicular to the surface) into the spinal cord tissue; this cannula was advanced 1 mm so minipump flow would directly reach the ventral horn. Cannulas were built with a hollow glass filament 2 mm in length (internal diameter, 50 μm; external diameter, 80 μm; VitroCom) inserted into plastic flexible tubing ∼1.5 cm long. Implants were made by pouring dental acrylic cement onto the lamina, screw and cannula in order to fix them in place. Osmotic minipumps were connected to the cannula and placed subcutaneously in the back of the rat. Finally, the skin incision was closed with surgical stainless-steel clips and the rats received a single introperitoneal antibiotic dose (5% enrofloxacin; Baytril, Bayer). Once recovered from anesthesia, animals were monitored to verify that there were no motor alterations secondary to the surgical procedure. Rats were kept in individual cages and maintained as previously described for 7 or 10 days after surgery until sacrifice.

### Behavior evaluation

Rats were trained in two motor behavioral tests, rotarod and hindlimb strength assessment, 4-5 days before surgery. Rotarod (Columbus Instrument) assesses an animal's ability to walk on top of an accelerating roller at 2 rpm/s from 10 rpm/s for 120 s; the longest time to fall is recorded out of three attempts. Hindlimb strength was measured with a grip strength meter (TSE Systems) by placing the animal's hind paws on the grid and gently stimulating the animal's tail to elicit a forward jump into a black box placed in front of them; care was taken to prevent the animals from using their forelimbs during the forward jump. The greatest strength, as measured in pounds, out of three attempts was recorded and normalized to basal values (individual value of each rat obtained immediately before surgery, day 0). These behavioral tests were performed daily, starting 24 h after surgery, for 7 or 10 days depending on the experimental groups, always before i.p. treatment administration (NaBut or vehicle), and until sacrifice.

### Muscle and spinal cord histology and immunofluorescence

In order to quantify spinal MNs, animals were sacrificed with a lethal dose of pentobarbital administered intraperitoneally. Before cardiac arrest, animals were transcardially perfused with 250 ml of 0.9% saline solution followed by 250 ml of 4% paraformaldehyde in PBS pH 7.4. Cannula permeability was meticulously checked after perfusion, and 1 cm of spinal cord tissue around the infusion site was collected. Spinal cord tissue was post-fixed for 48 h and then dehydrated with 30% sucrose in PBS for 24-48 h. Transverse sections, 40 μm thick, of the spinal cord were obtained in a cryostat, of which 20 slices (with visual evidence of cannula insertion) were mounted in gelatinized glass slides and stained with Cresyl Violet (Nissl stain). The numbers of healthy MNs in the ventral horns were counted, ipsilateral and contralateral to the infusion side. An MN was considered healthy if the soma size was >25 μm, it had clear cytoplasm, the nucleus was visible and it had defined neurite projections.

Fresh biceps femoris muscle (ipsilateral and contralateral) tissue was also collected from vehicle- and AMPA-infused animals. For Hematoxylin and Eosin staining, muscle samples were fixed in 10% formalin in PBS for 4 h, then transversal 7 μm sections were obtained from paraffin-embedded tissue, and, following deparaffinization, the tissues were incubated in Hematoxylin for 5 min, washed with water and 100% ethanol, incubated in Eosin for 30 s, and thoroughly washed with ethanol and xylene solutions. Samples were then covered with Permount (Fisher Scientific) and coated with a glass coverslip. To visualize the NMJ, tissue was fixed in 2% paraformaldehyde in PBS for 24 h and cryopreserved in 30% sucrose for 24 h, then longitudinal 35 μm sections were obtained in a cryostat. Sections were incubated with 0.1 M glycine/PBS for 30 min for aldehyde block and permeabilized methanol for 7 min. Blocking was performed with PBS 0.1 M/0.2% v/v Triton X-100/2% w/v bovine serum albumin for 60 min; all subsequent procedures were carried out with this solution but with no albumin. Tissue sections were incubated with a cocktail of primary antibodies at 4.0°C for 48 h. Primary antibodies were used at the following dilutions: mouse anti-synaptophysin (Syp; Santa Cruz Biotechnology), 1:20; mouse anti-SMI 311 and anti-SMI 312 (Abcam), 1:2000. Later, primary antibodies were washed three times, and sections were sequentially incubated with (1) Alexa Fluor (AF) 488-conjugated ɑ-bungarotoxin 3 μg/ml and (2) donkey anti-mouse IgG AF546 (Life Technologies) for 120 min in the dark and at room temperature, and then were washed three times before placing the coverslips with simple fluorescent mounting medium (Dako). Fluorescence imaging was performed in a Zeiss LSM 800 confocal microscope. Imaging parameters (laser intensity, gain, digital offset, confocal aperture) were manually adjusted. Stacks were obtained by composing captured images every 1 μm that spanned the complete thickness of the tissue, with a 20× objective, of the selected field composed of two channels: green for ɑ-bungarotoxin/AF488 imaging and red for Syp-SMI311-SMI312/AF546 imaging. Maximal-intensity projections, merged images and digital zoom were obtained offline with the FIJI program.

### Cellular fractionation and western blotting

Animals were decapitated to collect fresh ipsilateral and contralateral biceps femoris muscle tissue. This muscle was selected because it is highly innervated by MNs from the L4 lumbar segment (Mohan et al., 2015) and it is easily accessible.

Cellular fractionation was performed as described by Dimauro et al. (2012) with minor modifications. Muscle tissues were quickly dissected and homogenized (OMNI Tissue Homogenizer TH, OMNI International) in 500 μl STM buffer [250 mM sucrose, 50 mM Tris-HCl pH 7.4, 5 mM MgCl_2_, cOmplete protease inhibitor cocktail (Roche), phenylmethylsulfonyl fluoride 1 mM]. The homogenate was vortexed for 15 s and centrifuged at 800 ***g*** for 15 min. The supernatant was decanted into a centrifuge tube (cytosolic fraction), and the pellet was resuspended in 500 μl STM buffer (nuclear fraction). The supernatant was then centrifuged twice for 10 min at 800 ***g*** and 11,000 ***g***, respectively, and the resulting supernatant, the cytosolic fraction, was collected and saved for further analysis. The resuspended pellet was vortexed for 15 s, and then centrifugation (500 ***g*** for 15 min) and washing were done twice to discard any remaining cytosolic remnants. The resuspended pellet was centrifuged at 1000 ***g*** for 15 min, and the resulting pellet was resuspended in 500 μl NET buffer [20 mM HEPES pH 7.9, 1.5 mM MgCl_2_, 0.5 M NaCl, 0.2 mM EDTA, 20% glycerol, 1% Triton X-100, cOmplete protease inhibitor cocktail (Roche), phenylmethylsulfonyl fluoride 1 mM], vortexed for 15 s, and sonicated (Branson Sonifier 250, Branson Ultrasonics) at 4 Hz for 15 s while on ice. The homogenate was centrifuged at 9000 ***g*** for 30 min (at 4°C), and the resulting supernatant was the nuclear fraction.

For whole-tissue blotting, biceps femoris muscles were quickly dissected and homogenized in lysis buffer [50 mM Tris-base pH 8, 150 mM NaCl, 1% Triton X-100, 0.5% sodium deoxycholate, 1% SDS, 2 mg/ml cOmplete protease inhibitor cocktail (Roche), 1 mM phenylmethylsulfonyl fluoride], and centrifuged at 15,700 ***g***.

A small sample (5 μl) of homogenates was taken to measure protein concentration by the Lowry modified method (DC protein assay, Bio-Rad). The remaining homogenate was heat denatured (100°C for 10 min) with a 1:1 2× Laemmli sample buffer solution and stored at −70°C. For protein immunodetection, 20 µg tissue protein extract was loaded into acrylamide mini-gels and transferred onto PVDF membranes. Care was taken to always load comparable tissue protein extract according to the sides of spinal infusions (ipsilateral or contralateral) in the same acrylamide gel, in order to perform comparisons only relating to treatment and not to other effects, such a cannula insertion (as observed by Lazo-Gomez and Tapia, 2017). The following primary antibodies were used in this study: anti-HDAC4 (1:1500; Abcam), anti-acetylated histone 3 at lysines 9 and 14 (H3K9,14) (1:1000; Santa Cruz Biotechnology), anti-GAPDH (1:6000; GeneTex) and anti-lamin B (1:1000; Abcam). Then, membranes were washed three times for 10 min and incubated with the following secondary antibodies: horseradish peroxidase (HRP)-conjugated goat anti-rabbit IgG (1:5000; Cell Signaling Technology), HRP-conjugated goat anti-mouse IgG (1:10,000; Jackson ImmunoResearch) and IRDye^®^ 800CW goat anti-mouse IgG (1:5000; LI-COR Biosciences). Chemiluminescence substrates used were Luminata Forte Western HRP (Merck) and Western Blotting Luminol Reagent (Santa Cruz Biotechnology), and immunoreactivity was detected with a C-DiGit Blot Scanner (LI-COR Biosciences). Immunofluorescence was used only for GAPDH, with detection was performed in an Odyssey CLx Imager (LI-COR Biosciences). Band intensity was determined using FIJI software and normalized to the corresponding vehicle-treated group.

### Statistical analysis

Statistical analyses were performed in Prism 7 (GraphPad Software, San Diego, CA, USA). The results from western blotting, behavioral tests and MN counting were expressed as means±s.e.m. One-way analysis of variance (ANOVA) with Bonferroni's post-hoc test were performed to compare means between groups of the results obtained from western blotting and MN counting. Behavioral test results were compared with two-way ANOVA (where the factors were the assigned treatment group and time) with Bonferroni's post-hoc analyses. For all statistical tests, *P*<0.05 was considered significant.

## Supplementary Material

10.1242/dmm.049851_sup1 Supplementary information
