# Supplementary material for: Sodium butyrate does not protect spinal motor neurons from AMPA-induced excitotoxic degeneration in vivo
Source: Dis Model Mech. 2023 Oct 13;16(10):dmm049851. doi: 10.1242/dmm.049851 (PMC10581382; doi:10.1242/dmm.049851)
Supplement: Supplementary information [file dmm-16-049851-s1.pdf]

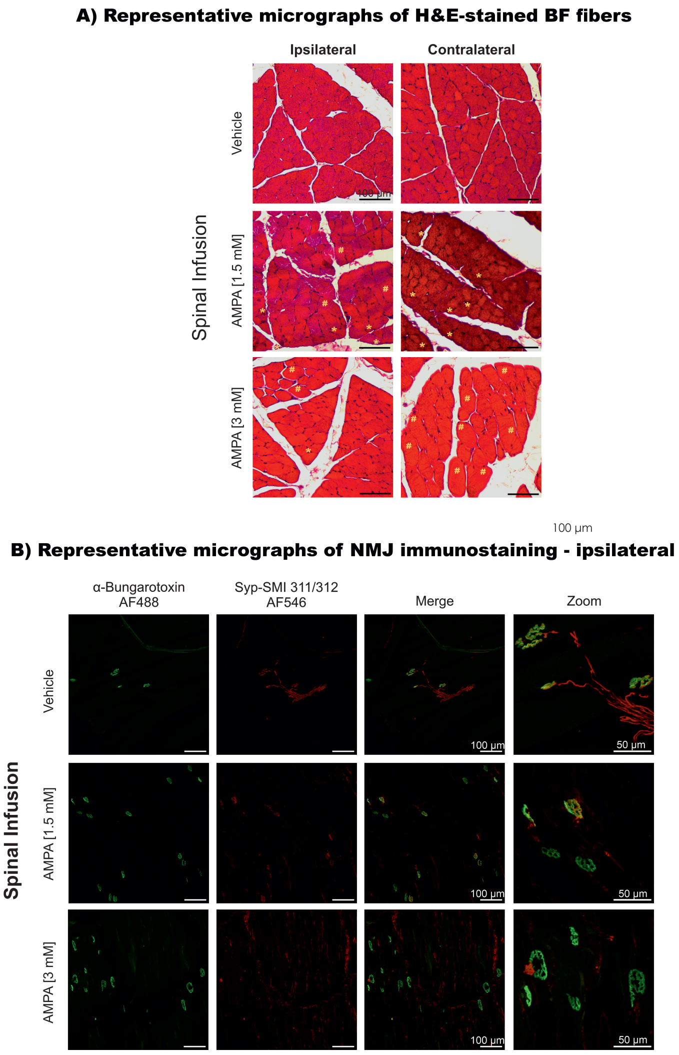


**Fig. S1. Biceps femoris muscle atrophy and denervation induced by chronic spinal excitotoxicity.** Representative micrographs of H&E-stained transversal ipsilateral and contralateral biceps femorii muscle fibers displaying both atrophy (*) and compensatory hypertrophy (#) (**A**), and representative immunofluorescent maximal intensity projection stack of confocal micrographs of NMJs pre- (red channel, synaptophysin –Syp– and heavy neurofilaments –SMI311/312) and post-synapsis (green channel, α-bungarotoxin) in the ipsilateral biceps femorii from animals spinally infused with vehicle (upper row), AMPA 1.5 mM for 10 days (middle row) or AMPA 3 mM for 7 days (bottom row) (**B**).
